# Supplementary material for: Tuberculin skin test and Interferon-gamma release assay agreement, and associated factors with latent tuberculosis infection, in medical and nursing students in Bandung, Indonesia
Source: PLoS One. 2024 Mar 18;19(3):e0299874. doi: 10.1371/journal.pone.0299874 (PMC10947906; doi:10.1371/journal.pone.0299874)
Supplement: S1 Dataset — (PDF) [file pone.0299874.s001.pdf]

| Study ID | Age | Gender | Student type | Housing | Ethnicity | BCG vac | Smoking | Alcohol | Training | TB contact | Family or friend contact | BMI | BMI category | IGRA result | TST result | TST induration | IGRA (TB1-Nil) |
|---|---|---|---|---|---|---|---|---|---|---|---|---|---|---|---|---|---|
| 1401143 | 21,54 | 1 | 0 | 0 | 1 | 1 | 0 | 0 | 1 | 1 | 0 | 29,8 | 3 | 1 | 1 | 11 | 0,77 |
| 1401144 | 21,90 | 0 | 0 | 0 | 2 | 1 | 0 | 0 | 1 | 1 | 0 | 26,5 | 3 | 0 | 0 | 0 | 0,01 |
| 1401145 | 22,83 | 1 | 0 | 0 | 1 | 1 | 1 | 1 | 1 | 0 | 1 | 25,6 | 3 | 0 | 0 | 0 | 0,05 |
| 1401146 | 22,39 | 0 | 0 | 0 | 1 | 0 | 0 | 0 | 0 | 0 | 1 | 20,5 | 2 | 0 | 0 | 0 | -0,05 |
| 1401147 | 21,27 | 0 | 0 | 1 | 1 | 0 | 0 | 0 | 1 | 1 | 0 | 22,0 | 2 | 0 | 0 | 9 | -0,09 |
| 1401148 | 23,74 | 0 | 0 | 0 | 2 | 1 | 0 | 1 | 1 | 1 | 0 | 27,9 | 3 | 0 | 0 | 0 | 0,21 |
| 1401149 | 22,64 | 0 | 0 | 0 | 2 | 1 | 0 | 1 | 1 | 0 | 0 | 28,4 | 3 | 0 | 0 | 9 | 0,05 |
| 1401150 | 22,92 | 0 | 0 | 0 | 2 | 1 | 0 | 0 | 0 | 0 | 0 | 21,9 | 2 | 0 | 1 | 16 | 0,07 |
| 1401151 | 23,98 | 0 | 0 | 0 | 1 | 1 | 0 | 0 | 1 | 1 | 0 | 21,3 | 2 | 0 | 0 | 4 | -0,3 |
| 1401152 | 21,89 | 1 | 0 | 1 | 1 | 1 | 0 | 0 | 0 | 0 | 0 | 35,5 | 4 | 0 | 0 | 5 | 0 |
| 1401153 | 21,59 | 1 | 0 | 1 | 1 | 0 | 0 | 0 | 0 | 0 | 0 | 28,7 | 3 | 0 | 1 | 13 | 0 |
| 1401154 | 22,17 | 0 | 0 | 0 | 2 | 1 | 0 | 0 | 0 | 0 | 0 | 28,9 | 3 | 0 | 1 | 12 | 0,02 |
| 1401155 | 24,97 | 0 | 0 | 0 | 2 | 1 | 0 | 0 | 0 | 0 | 0 | 28,4 | 3 | 0 | 1 | 11 | -0,67 |
| 1401156 | 28,57 | 0 | 0 | 0 | 2 | 1 | 0 | 0 | 1 | 1 | 0 | 21,9 | 2 | 0 | 1 | 23 | -0,04 |
| 1401157 | 21,35 | 0 | 0 | 1 | 2 | 1 | 0 | 0 | 1 | 1 | 1 | 21,7 | 2 | 0 | 0 | 6 | -0,13 |
| 1401158 | 20,27 | 1 | 0 | 0 | 2 | 0 | 0 | 0 | 0 | 0 | 0 | 22,2 | 2 | 0 | 0 | 7 | 0 |
| 1401159 | 22,20 | 1 | 0 | 0 | 2 | 1 | 1 | 0 | 1 | 0 | 1 | 24,1 | 2 | 0 | 1 | 11 | -0,01 |
| 1401160 | 21,85 | 0 | 0 | 1 | 1 | 1 | 0 | 0 | 1 | 0 | 0 | 23,4 | 2 | 0 | 0 | 0 | -0,01 |
| 1401161 | 22,52 | 0 | 0 | 0 | 2 | 1 | 0 | 0 | 1 | 0 | 1 | 20,4 | 2 | 0 | 0 | 2 | 0 |
| 1401162 | 23,61 | 0 | 0 | 0 | 2 | 1 | 0 | 0 | 0 | 0 | 0 | 24,3 | 2 | 1 | 0 | 0 | 1,66 |
| 1401163 | 21,29 | 1 | 0 | 0 | 2 | 1 | 1 | 0 | 0 | 0 | 0 | 29,7 | 3 | 0 | 0 | 9 | 0,07 |
| 1401164 | 24,04 | 1 | 0 | 0 | 2 | 1 | 1 | 0 | 1 | 1 | 0 | 19,5 | 2 | 0 | 1 | 12 | 0,14 |
| 1401165 | 25,37 | 1 | 0 | 0 | 2 | 1 | 0 | 1 | 1 | 1 | 0 | 26,5 | 3 | 1 | 1 | 15 | 1 |
| 1401166 | 21,56 | 0 | 0 | 0 | 2 | 1 | 0 | 0 | 1 | 0 | 0 | 18,8 | 2 | 0 | 0 | 0 | 0,02 |
| 1401167 | 24,12 | 0 | 0 | 0 | 2 | 1 | 0 | 0 | 1 | 0 | 0 | 31,0 | 4 | 0 | 0 | 0 | 0,02 |
| 1401168 | 21,62 | 0 | 0 | 0 | 1 | 1 | 0 | 0 | 1 | 0 | 0 | 26,3 | 3 | 1 | 1 | 20 | 0,57 |
| 1401169 | 21,41 | 0 | 0 | 0 | 2 | 1 | 0 | 0 | 1 | 0 | 1 | 22,4 | 2 | 0 | 0 | 2 | -0,06 |
| 1401170 | 21,34 | 0 | 0 | 0 | 2 | 1 | 0 | 0 | 1 | 1 | 1 | 25,5 | 3 | 0 | 0 | 9 | -0,01 |
| 1401171 | 22,95 | 0 | 0 | 0 | 2 | 1 | 0 | 0 | 1 | 0 | 0 | 16,3 | 1 | 0 | 0 | 1 | -0,08 |
| 1401172 | 22,28 | 0 | 0 | 0 | 2 | 1 | 0 | 1 | 1 | 0 | 0 | 18,5 | 1 | 0 | 1 | 15 | -0,01 |
| 1401173 | 22,50 | 0 | 0 | 0 | 2 | 1 | 0 | 0 | 0 | 0 | 1 | 22,1 | 2 | 1 | 1 | 19 | 0,53 |
| 1401174 | 21,37 | 1 | 0 | 0 | 1 | 1 | 0 | 0 | 1 | 0 | 0 | 21,5 | 2 | 0 | 0 | 0 | 0 |
| 1401175 | 22,18 | 0 | 0 | 0 | 2 | 1 | 0 | 0 | 0 | 0 | 0 | 17,9 | 1 | 0 | 0 | 5 | 0,05 |
| 1401176 | 23,31 | 0 | 0 | 0 | 2 | 1 | 0 | 1 | 0 | 0 | 0 | 26,9 | 3 | 0 | 0 | 8 | 0 |
| 1401177 | 22,52 | 0 | 0 | 0 | 2 | 1 | 0 | 0 | 1 | 0 | 0 | 36,3 | 4 | 1 | 0 | 6 | 0,05 |
| 1401178 | 20,90 | 0 | 0 | 1 | 1 | 1 | 0 | 0 | 0 | 0 | 1 | 18,7 | 2 | 0 | 0 | 2 | -0,01 |
| 1401179 | 21,23 | 0 | 0 | 0 | 1 | 1 | 0 | 0 | 0 | 0 | 0 | 26,9 | 3 | 0 | 1 | 19 | 0,06 |
| 1401180 | 20,49 | 1 | 0 | 1 | 2 | 1 | 0 | 0 | 0 | 0 | 0 | 22,1 | 2 | 0 | 0 | 2 | 0 |
| 1401181 | 22,66 | 0 | 0 | 0 | 2 | 1 | 0 | 0 | 1 | 1 | 0 | 21,7 | 2 | 0 | 0 | 7 | 0 |
| 1401182 | 23,92 | 1 | 0 | 0 | 1 | 1 | 1 | 0 | 0 | 0 | 0 | 24,7 | 2 | 0 | 1 | 12 | 0,04 |
| 1401183 | 20,88 | 1 | 0 | 0 | 2 | 1 | 1 | 0 | 0 | 0 | 0 | 24,5 | 2 | 0 | 0 | 3 | 0 |

| | | | | | | | | | | | | | | | | | |
|---|---|---|---|---|---|---|---|---|---|---|---|---|---|---|---|---|---|
| 1401184 | 22,99 | 1 | 0 | 0 | 1 | 1 | 0 | 0 | 0 | 0 | 0 | 21,8 | 2 | 1 | 1 | 10 | 0,55 |
| 1401185 | 22,06 | 1 | 0 | 0 | 1 | 1 | 1 | 0 | 0 | 0 | 0 | 21,3 | 2 | 0 | 0 | 9 | 0,01 |
| 1401186 | 20,59 | 1 | 0 | 0 | 2 | 1 | 0 | 0 | 0 | 0 | 0 | 22,8 | 2 | 0 | 0 | 0 | 0,03 |
| 1401187 | 20,68 | 0 | 0 | 0 | 1 | 1 | 0 | 0 | 1 | 1 | 0 | 28,3 | 3 | 0 | 1 | 13 | -0,36 |
| 1401188 | 21,14 | 0 | 0 | 0 | 1 | 1 | 0 | 0 | 1 | 1 | 0 | 23,5 | 2 | 1 | 1 | 11 | 3,32 |
| 1401189 | 22,14 | 0 | 0 | 0 | 1 | 1 | 0 | 0 | 0 | 0 | 0 | 21,9 | 2 | 0 | 0 | 5 | -0,01 |
| 1401190 | 22,29 | 1 | 0 | 0 | 2 | 1 | 1 | 1 | 0 | 1 | 0 | 28,7 | 3 | 1 | 0 | 0 | 0,38 |
| 1401191 | 22,98 | 1 | 0 | 0 | 2 | 1 | 0 | 0 | 0 | 0 | 0 | 28,5 | 3 | 0 | 0 | 0 | 0 |
| 1401192 | 23,14 | 0 | 0 | 0 | 1 | 1 | 1 | 1 | 0 | 0 | 0 | 26,4 | 3 | 0 | 0 | 2 | -0,02 |
| 1401193 | 22,28 | 1 | 0 | 1 | 1 | 1 | 1 | 1 | 0 | 0 | 0 | 16,2 | 1 | 0 | 0 | 9 | 0,02 |
| 1401194 | 21,86 | 1 | 0 | 0 | 2 | 1 | 0 | 0 | 1 | 1 | 0 | 25,7 | 3 | 0 | 1 | 13 | 0 |
| 1401195 | 21,15 | 0 | 0 | 0 | 2 | 1 | 0 | 0 | 1 | 1 | 0 | 16,5 | 1 | 0 | 0 | 0 | 0 |
| 1401196 | 23,80 | 0 | 0 | 0 | 2 | 1 | 0 | 1 | 1 | 0 | 0 | 30,0 | 3 | 0 | 1 | 15 | -0,01 |
| 1401197 | 25,77 | 0 | 0 | 0 | 2 | 1 | 0 | 0 | 0 | 0 | 0 | 23,9 | 2 | 0 | 0 | 0 | 0,07 |
| 1401198 | 22,69 | 0 | 0 | 0 | 2 | 1 | 0 | 0 | 1 | 0 | 0 | 17,7 | 1 | 0 | 0 | 7 | 0 |
| 1401199 | 22,01 | 0 | 0 | 0 | 2 | 1 | 0 | 0 | 1 | 1 | 0 | 22,7 | 2 | 1 | 1 | 14 | 1,02 |
| 1401200 | 23,41 | 0 | 0 | 0 | 2 | 1 | 0 | 1 | 1 | 0 | 0 | 20,1 | 2 | 0 | 1 | 11 | -0,01 |
| 1401201 | 23,07 | 0 | 0 | 0 | 2 | 1 | 0 | 0 | 1 | 1 | 0 | 23,6 | 2 | 0 | 0 | 0 | 0,02 |
| 1401202 | 24,09 | 1 | 0 | 0 | 2 | 1 | 0 | 0 | 1 | 1 | 0 | 27,6 | 3 | 0 | 0 | 6 | -0,01 |
| 1401203 | 23,46 | 0 | 0 | 0 | 2 | 1 | 0 | 1 | 1 | 0 | 0 | 17,2 | 1 | 1 | 0 | 6 | 0,14 |
| 1401204 | 21,19 | 0 | 0 | 0 | 2 | 1 | 0 | 0 | 1 | 1 | 0 | 20,9 | 2 | 1 | 0 | 0 | 1,83 |
| 1401205 | 20,06 | 1 | 0 | 0 | 2 | 1 | 0 | 0 | 1 | 0 | 1 | 27,3 | 3 | 0 | 1 | 18 | 0 |
| 1401206 | 20,83 | 0 | 0 | 0 | 2 | 0 | 0 | 0 | 1 | 1 | 0 | 23,0 | 2 | 0 | 0 | 9 | -0,01 |
| 1401207 | 22,32 | 0 | 0 | 0 | 2 | 1 | 0 | 0 | 1 | 0 | 0 | 19,6 | 2 | 0 | 1 | 10 | 0 |
| 1401208 | 23,87 | 1 | 0 | 0 | 2 | 1 | 1 | 1 | 1 | 0 | 0 | 28,1 | 3 | 0 | 0 | 6 | 0,05 |
| 1401209 | 21,48 | 0 | 0 | 0 | 2 | 1 | 0 | 0 | 1 | 1 | 0 | 21,6 | 2 | 0 | 0 | 0 | -0,05 |
| 1401210 | 23,50 | 0 | 0 | 0 | 2 | 1 | 0 | 0 | 0 | 0 | 0 | 27,5 | 3 | 0 | 0 | 2 | 0 |
| 1401211 | 25,15 | 0 | 0 | 0 | 2 | 1 | 0 | 0 | 0 | 0 | 0 | 28,4 | 3 | 0 | 0 | 2 | 0 |
| 1401212 | 21,99 | 1 | 0 | 0 | 2 | 1 | 1 | 0 | 1 | 1 | 0 | 29,2 | 3 | 0 | 1 | 12 | 0,03 |
| 1401213 | 21,47 | 0 | 0 | 0 | 2 | 1 | 0 | 0 | 0 | 0 | 0 | 16,3 | 1 | 1 | 1 | 10 | 0,46 |
| 1401214 | 21,29 | 0 | 0 | 0 | 2 | 1 | 0 | 0 | 1 | 1 | 0 | 20,1 | 2 | 0 | 1 | 19 | 0 |
| 1401215 | 21,84 | 0 | 0 | 1 | 1 | 1 | 0 | 0 | 1 | 1 | 0 | 21,5 | 2 | 0 | 0 | 2 | -0,01 |
| 1401216 | 21,42 | 0 | 0 | 0 | 2 | 1 | 0 | 0 | 1 | 1 | 0 | 15,6 | 1 | 0 | 0 | 3 | 0,06 |
| 1401217 | 21,27 | 0 | 0 | 0 | 1 | 0 | 0 | 0 | 1 | 0 | 0 | 17,8 | 1 | 0 | 0 | 1 | -0,02 |
| 1401218 | 20,77 | 0 | 0 | 0 | 2 | 1 | 0 | 0 | 1 | 1 | 0 | 22,1 | 2 | 0 | 0 | 0 | 0,01 |
| 1401219 | 21,67 | 0 | 0 | 0 | 1 | 1 | 0 | 0 | 1 | 0 | 0 | 20,5 | 2 | 1 | 1 | 25 | 2,45 |
| 1401220 | 21,59 | 0 | 0 | 0 | 2 | 1 | 0 | 0 | 1 | 1 | 0 | 21,7 | 2 | 1 | 1 | 20 | 0,65 |
| 1401221 | 21,63 | 0 | 0 | 1 | 2 | 1 | 0 | 0 | 1 | 1 | 0 | 19,7 | 2 | 0 | 1 | 10 | 0,02 |
| 1401222 | 21,19 | 0 | 0 | 0 | 1 | 1 | 0 | 0 | 1 | 0 | 1 | 23,3 | 2 | 0 | 0 | 0 | 0,05 |
| 1401223 | 21,97 | 0 | 0 | 0 | 2 | 1 | 0 | 0 | 1 | 0 | 1 | 20,9 | 2 | 0 | 0 | 8 | 0,21 |
| 1401224 | 21,28 | 0 | 0 | 0 | 2 | 1 | 0 | 0 | 1 | 0 | 1 | 26,9 | 3 | 0 | 0 | 0 | 0,02 |
| 1401225 | 22,59 | 1 | 0 | 0 | 1 | 1 | 0 | 1 | 1 | 0 | 0 | 29,1 | 3 | 0 | 1 | 17 | 0,18 |
| 1401226 | 22,39 | 1 | 0 | 0 | 1 | 1 | 0 | 1 | 1 | 0 | 0 | 18,4 | 1 | 0 | 1 | 11 | 0,01 |
| 1401227 | 23,48 | 0 | 0 | 0 | 2 | 1 | 0 | 0 | 0 | 0 | 0 | 22,6 | 2 | 0 | 0 | 9 | 0,01 |

| | | | | | | | | | | | | | | | | | |
|---|---|---|---|---|---|---|---|---|---|---|---|---|---|---|---|---|---|
| 1401228 | 21,88 | 0 | 0 | 0 | 2 | 1 | 0 | 0 | 1 | 0 | 0 | 19,9 | 2 | 0 | 1 | 14 | -0,05 |
| 1401229 | 22,16 | 1 | 0 | 0 | 2 | 1 | 0 | 0 | 1 | 1 | 0 | 22,8 | 2 | 0 | 0 | 0 | -0,04 |
| 1401230 | 22,28 | 1 | 0 | 0 | 2 | 0 | 0 | 0 | 1 | 0 | 0 | 27,1 | 3 | 0 | 0 | 9 | 0,01 |
| 1401231 | 21,54 | 0 | 0 | 0 | 2 | 0 | 0 | 0 | 1 | 0 | 0 | 18,5 | 1 | 0 | 1 | 19 | 0,03 |
| 1401232 | 23,28 | 0 | 0 | 0 | 1 | 1 | 0 | 0 | 0 | 0 | 1 | 21,9 | 2 | 0 | 0 | 5 | 0,17 |
| 1401233 | 21,51 | 0 | 0 | 0 | 2 | 1 | 0 | 1 | 1 | 1 | 0 | 17,9 | 1 | 0 | 0 | 3 | 0,03 |
| 1401234 | 21,86 | 0 | 0 | 0 | 2 | 1 | 0 | 0 | 1 | 0 | 0 | 20,5 | 2 | 0 | 0 | 2 | 0 |
| 1401235 | 21,53 | 0 | 0 | 1 | 2 | 1 | 0 | 0 | 1 | 1 | 1 | 19,9 | 2 | 0 | 1 | 11 | -0,02 |
| 1401236 | 21,10 | 0 | 0 | 0 | 2 | 1 | 0 | 0 | 1 | 1 | 0 | 20,0 | 2 | 0 | 0 | 4 | 0 |
| 1401237 | 20,91 | 0 | 0 | 0 | 2 | 1 | 0 | 0 | 1 | 0 | 1 | 24,2 | 2 | 0 | 0 | 2 | 0,07 |
| 1401238 | 21,75 | 0 | 0 | 0 | 1 | 1 | 0 | 0 | 1 | 0 | 0 | 24,7 | 2 | 1 | 0 | 0 | 0,43 |
| 1401239 | 21,14 | 0 | 0 | 0 | 2 | 1 | 0 | 0 | 1 | 1 | 0 | 19,4 | 2 | 1 | 1 | 16 | 7,09 |
| 1401240 | 21,92 | 0 | 0 | 0 | 1 | 0 | 0 | 0 | 1 | 1 | 0 | 26,8 | 3 | 0 | 0 | 0 | 0 |
| 1401241 | 20,61 | 0 | 0 | 0 | 1 | 1 | 0 | 0 | 1 | 0 | 1 | 20,5 | 2 | 0 | 0 | 8 | 0,01 |
| 1401242 | 20,27 | 0 | 0 | 0 | 2 | 0 | 0 | 0 | 0 | 0 | 0 | 28,6 | 3 | 1 | 1 | 14 | 0,86 |
| 1401243 | 21,66 | 0 | 0 | 0 | 1 | 1 | 0 | 0 | 0 | 0 | 0 | 22,2 | 2 | 0 | 0 | 6 | -0,01 |
| 1401244 | 21,10 | 0 | 0 | 0 | 2 | 1 | 0 | 0 | 1 | 1 | 0 | 27,0 | 3 | 0 | 0 | 4 | 0 |
| 1401245 | 21,63 | 0 | 0 | 0 | 1 | 0 | 0 | 0 | 1 | 1 | 1 | 20,2 | 2 | 0 | 1 | 43 | 0,23 |
| 1401246 | 21,41 | 0 | 0 | 0 | 2 | 1 | 0 | 1 | 1 | 0 | 0 | 21,1 | 2 | 0 | 1 | 16 | -0,07 |
| 1401247 | 21,87 | 0 | 0 | 0 | 2 | 1 | 0 | 0 | 0 | 0 | 0 | 26,1 | 3 | 0 | 0 | 0 | -0,02 |
| 1401248 | 22,05 | 0 | 0 | 0 | 2 | 1 | 0 | 0 | 1 | 0 | 0 | 21,4 | 2 | 0 | 0 | 0 | -0,03 |
| 1401249 | 21,61 | 0 | 0 | 0 | 2 | 0 | 1 | 0 | 1 | 0 | 0 | 25,9 | 3 | 0 | 0 | 7 | 0,01 |
| 1401250 | 22,21 | 0 | 0 | 0 | 1 | 1 | 0 | 0 | 1 | 1 | 1 | 21,6 | 2 | 0 | 0 | 4 | 0,02 |
| 1401251 | 20,86 | 1 | 0 | 0 | 2 | 1 | 1 | 0 | 1 | 1 | 0 | 23,0 | 2 | 0 | 0 | 6 | 0 |
| 1401252 | 21,29 | 1 | 0 | 0 | 2 | 1 | 0 | 0 | 1 | 1 | 0 | 35,8 | 4 | 0 | 0 | 0 | 0,07 |
| 1401253 | 22,22 | 1 | 0 | 0 | 2 | 1 | 0 | 0 | 0 | 0 | 0 | 22,1 | 2 | 0 | 0 | 0 | 0 |
| 1401254 | 21,54 | 1 | 0 | 1 | 2 | 1 | 0 | 0 | 1 | 1 | 0 | 20,7 | 2 | 0 | 0 | 0 | 0,01 |
| 1401255 | 22,88 | 0 | 0 | 1 | 2 | 1 | 0 | 0 | 1 | 1 | 0 | 17,5 | 1 | 0 | 0 | 2 | 0,13 |
| 1401256 | 21,95 | 0 | 0 | 0 | 2 | 1 | 0 | 0 | 1 | 1 | 0 | 20,7 | 2 | 0 | 0 | 0 | 0,02 |
| 1401257 | 19,80 | 0 | 0 | 1 | 1 | 1 | 0 | 0 | 1 | 1 | 0 | 22,9 | 2 | 0 | 0 | 0 | -0,01 |
| 1401258 | 20,31 | 0 | 0 | 0 | 2 | 1 | 0 | 0 | 1 | 1 | 0 | 22,5 | 2 | 1 | 0 | 0 | 0,86 |
| 1401259 | 19,96 | 0 | 0 | 1 | 1 | 0 | 0 | 0 | 1 | 1 | 1 | 19,4 | 2 | 0 | 0 | 6 | -0,01 |
| 1401260 | 21,05 | 0 | 0 | 0 | 1 | 1 | 0 | 0 | 1 | 1 | 0 | 22,8 | 2 | 0 | 0 | 7 | 0,09 |
| 1401261 | 21,76 | 0 | 0 | 0 | 2 | 1 | 0 | 0 | 1 | 1 | 0 | 29,9 | 3 | 0 | 1 | 14 | -0,01 |
| 1401262 | 21,15 | 0 | 0 | 0 | 2 | 1 | 0 | 0 | 1 | 1 | 0 | 19,6 | 2 | 0 | 0 | 0 | 0 |
| 1401263 | 21,61 | 0 | 0 | 1 | 2 | 1 | 0 | 0 | 1 | 1 | 0 | 23,8 | 2 | 0 | 0 | 2 | -0,03 |
| 1401264 | 19,95 | 0 | 0 | 0 | 1 | 1 | 0 | 0 | 1 | 1 | 0 | 27,0 | 3 | 0 | 0 | 0 | 0 |
| 1401265 | 21,54 | 0 | 0 | 0 | 2 | 1 | 0 | 0 | 1 | 0 | 0 | 24,1 | 2 | 0 | 0 | 4 | -0,12 |
| 1401266 | 21,69 | 0 | 0 | 0 | 1 | 1 | 0 | 0 | 1 | 1 | 1 | 20,5 | 2 | 0 | 0 | 3 | 0,04 |
| 1401267 | 21,49 | 0 | 0 | 0 | 1 | 1 | 0 | 0 | 1 | 0 | 1 | 18,2 | 1 | 0 | 0 | 6 | 0,04 |
| 1401268 | 21,53 | 0 | 0 | 0 | 2 | 1 | 0 | 0 | 1 | 1 | 0 | 21,1 | 2 | 0 | 0 | 3 | 0,2 |
| 1401269 | 21,75 | 1 | 0 | 0 | 2 | 1 | 0 | 0 | 1 | 0 | 0 | 20,5 | 2 | 0 | 0 | 7 | -0,35 |
| 1401270 | 20,80 | 0 | 0 | 0 | 2 | 1 | 0 | 0 | 0 | 0 | 0 | 17,8 | 1 | 0 | 1 | 11 | 0,16 |
| 1401271 | 22,11 | 0 | 0 | 0 | 2 | 1 | 0 | 0 | 0 | 0 | 0 | 22,5 | 2 | 0 | 1 | 11 | -0,12 |

| | | | | | | | | | | | | | | | | | |
|---|---|---|---|---|---|---|---|---|---|---|---|---|---|---|---|---|---|
| 1401272 | 20,49 | 0 | 0 | 0 | 2 | 1 | 0 | 0 | 1 | 1 | 0 | 19,1 | 2 | 0 | 0 | 4 | 0,01 |
| 1401273 | 21,20 | 0 | 0 | 1 | 1 | 1 | 0 | 0 | 1 | 1 | 0 | 21,6 | 2 | 0 | 0 | 9 | -0,01 |
| 1401274 | 22,00 | 1 | 0 | 0 | 2 | 0 | 0 | 0 | 1 | 0 | 0 | 18,0 | 1 | 1 | 1 | 20 | 3,92 |
| 1401275 | 21,85 | 0 | 0 | 0 | 2 | 1 | 0 | 0 | 1 | 1 | 0 | 20,8 | 2 | 0 | 0 | 4 | -0,02 |
| 1401276 | 22,19 | 0 | 0 | 0 | 1 | 1 | 0 | 0 | 1 | 0 | 0 | 22,2 | 2 | 0 | 0 | 5 | -0,09 |
| 1401277 | 21,00 | 0 | 0 | 0 | 2 | 1 | 0 | 0 | 0 | 0 | 0 | 23,3 | 2 | 1 | 1 | 10 | -0,09 |
| 1401278 | 21,08 | 0 | 0 | 0 | 2 | 1 | 0 | 0 | 1 | 1 | 0 | 22,3 | 2 | 0 | 1 | 19 | 0,01 |
| 1401279 | 19,01 | 0 | 0 | 0 | 1 | 0 | 0 | 0 | 1 | 1 | 0 | 23,6 | 2 | 1 | 1 | 17 | 6,1 |
| 1401280 | 21,56 | 0 | 0 | 1 | 2 | 1 | 0 | 0 | 1 | 1 | 0 | 24,7 | 2 | 0 | 1 | 18 | 0,21 |
| 1401281 | 21,29 | 0 | 0 | 1 | 1 | 1 | 0 | 0 | 1 | 0 | 0 | 24,4 | 2 | 0 | 0 | 4 | 0 |
| 1401282 | 20,47 | 0 | 0 | 0 | 2 | 1 | 1 | 1 | 1 | 1 | 0 | 22,5 | 2 | 0 | 0 | 0 | 0 |
| 1401283 | 21,27 | 0 | 0 | 0 | 2 | 1 | 0 | 0 | 1 | 0 | 1 | 22,5 | 2 | 0 | 0 | 0 | 0,02 |
| 1401284 | 21,10 | 0 | 0 | 0 | 1 | 1 | 0 | 0 | 1 | 1 | 1 | 21,2 | 2 | 1 | 0 | 6 | 2,23 |
| 1401285 | 20,48 | 0 | 0 | 1 | 1 | 1 | 0 | 0 | 1 | 0 | 0 | 24,7 | 2 | 0 | 1 | 14 | 0 |
| 1401286 | 20,56 | 0 | 0 | 1 | 1 | 1 | 0 | 0 | 0 | 0 | 0 | 23,6 | 2 | 0 | 1 | 14 | 0 |
| 1401287 | 22,46 | 0 | 0 | 0 | 2 | 1 | 0 | 0 | 1 | 1 | 1 | 18,5 | 2 | 0 | 0 | 5 | 0,11 |
| 1401288 | 20,87 | 0 | 0 | 1 | 2 | 1 | 0 | 0 | 1 | 1 | 0 | 25,3 | 3 | 0 | 1 | 15 | 0,03 |
| 1401289 | 23,60 | 1 | 0 | 0 | 2 | 1 | 0 | 0 | 1 | 0 | 0 | 26,7 | 3 | 0 | 0 | 5 | 0,01 |
| 1401290 | 21,15 | 1 | 0 | 1 | 1 | 1 | 1 | 0 | 1 | 1 | 0 | 19,4 | 2 | 0 | 0 | 6 | -0,01 |
| 1401291 | 19,71 | 1 | 0 | 0 | 1 | 1 | 0 | 0 | 1 | 1 | 0 | 32,0 | 4 | 0 | 0 | 0 | 0,03 |
| 1401292 | 22,87 | 1 | 0 | 1 | 1 | 1 | 0 | 0 | 1 | 1 | 1 | 17,3 | 1 | 0 | 0 | 1 | -0,01 |
| 1401293 | 21,42 | 1 | 0 | 1 | 1 | 1 | 1 | 1 | 1 | 1 | 1 | 25,8 | 3 | 0 | 0 | 4 | 0,16 |
| 1401294 | 21,51 | 0 | 0 | 1 | 2 | 1 | 0 | 0 | 1 | 1 | 0 | 17,6 | 1 | 1 | 1 | 22 | 4,59 |
| 1401295 | 21,74 | 0 | 0 | 1 | 2 | 1 | 1 | 0 | 1 | 1 | 0 | 26,2 | 3 | 1 | 1 | 28 | 1,01 |
| 1401296 | 21,62 | 1 | 0 | 0 | 2 | 1 | 0 | 0 | 1 | 1 | 0 | 27,5 | 3 | 0 | 0 | 5 | -0,02 |
| 1401297 | 21,66 | 1 | 0 | 0 | 2 | 1 | 0 | 0 | 1 | 1 | 0 | 22,0 | 2 | 0 | 0 | 8 | -0,02 |
| 1401298 | 21,50 | 1 | 0 | 0 | 2 | 1 | 0 | 0 | 1 | 1 | 0 | 19,9 | 2 | 0 | 0 | 8 | -0,01 |
| 1401299 | 22,14 | 0 | 0 | 0 | 2 | 1 | 0 | 0 | 1 | 0 | 1 | 19,8 | 2 | 1 | 0 | 4 | 0,66 |
| 1401300 | 21,67 | 0 | 0 | 0 | 2 | 1 | 0 | 0 | 1 | 0 | 1 | 26,8 | 3 | 0 | 0 | 7 | -0,16 |
| 1401301 | 21,68 | 0 | 0 | 0 | 1 | 1 | 0 | 0 | 1 | 0 | 1 | 15,8 | 1 | 0 | 0 | 6 | -0,01 |
| 1401302 | 21,71 | 0 | 0 | 0 | 2 | 1 | 0 | 0 | 1 | 0 | 0 | 20,2 | 2 | 0 | 0 | 5 | 0,04 |
| 1401303 | 21,34 | 0 | 0 | 0 | 2 | 1 | 0 | 0 | 1 | 0 | 0 | 22,5 | 2 | 0 | 0 | 0 | -0,02 |
| 1401304 | 21,34 | 1 | 0 | 0 | 1 | 1 | 0 | 1 | 1 | 0 | 1 | 22,4 | 2 | 0 | 0 | 4 | 0,01 |
| 1401305 | 21,97 | 1 | 0 | 0 | 2 | 1 | 0 | 0 | 1 | 0 | 1 | 27,0 | 3 | 0 | 0 | 7 | 0,07 |
| 1401306 | 20,23 | 1 | 0 | 0 | 1 | 0 | 0 | 0 | 1 | 0 | 0 | 25,7 | 3 | 0 | 0 | 5 | 0,02 |
| 1401307 | 20,56 | 1 | 0 | 0 | 1 | 0 | 0 | 0 | 1 | 1 | 0 | 21,6 | 2 | 0 | 0 | 5 | 0,02 |
| 1401308 | 21,65 | 0 | 0 | 0 | 2 | 0 | 0 | 0 | 1 | 1 | 0 | 28,6 | 3 | 0 | 0 | 8 | -0,04 |
| 1401309 | 21,99 | 0 | 0 | 0 | 1 | 1 | 0 | 0 | 1 | 0 | 0 | 24,8 | 2 | 0 | 0 | 0 | 0,12 |
| 1401310 | 21,73 | 0 | 0 | 0 | 2 | 1 | 0 | 0 | 1 | 0 | 1 | 18,9 | 2 | 0 | 0 | 7 | 0,12 |
| 1401311 | 21,90 | 0 | 0 | 0 | 2 | 1 | 0 | 0 | 1 | 0 | 0 | 27,2 | 3 | 1 | 1 | 13 | 0,59 |
| 1401312 | 21,34 | 1 | 0 | 0 | 2 | 1 | 0 | 1 | 1 | 1 | 0 | 18,7 | 2 | 0 | 0 | 5 | 0,03 |
| 1401313 | 21,71 | 0 | 0 | 0 | 2 | 0 | 0 | 0 | 1 | 1 | 1 | 21,1 | 2 | 0 | 1 | 13 | 0,18 |
| 1401314 | 22,21 | 0 | 0 | 0 | 2 | 1 | 0 | 0 | 1 | 1 | 0 | 19,0 | 2 | 0 | 0 | 9 | 0,06 |
| 1401315 | 21,65 | 1 | 0 | 0 | 2 | 1 | 1 | 1 | 1 | 1 | 0 | 42,7 | 4 | 0 | 1 | 14 | -0,02 |

| | | | | | | | | | | | | | | | | | |
|---|---|---|---|---|---|---|---|---|---|---|---|---|---|---|---|---|---|
| 1401316 | 20,94 | 0 | 0 | 1 | 1 | 0 | 0 | 0 | 1 | 0 | 1 | 19,3 | 2 | 0 | 0 | 0 | 0 |
| 1401317 | 20,61 | 0 | 0 | 0 | 1 | 1 | 0 | 0 | 1 | 1 | 1 | 19,1 | 2 | 0 | 0 | 0 | 0,29 |
| 1401318 | 21,70 | 0 | 0 | 0 | 1 | 1 | 0 | 0 | 1 | 1 | 0 | 22,4 | 2 | 1 | 1 | 19 | 2,1 |
| 1401319 | 20,61 | 0 | 0 | 0 | 2 | 1 | 0 | 0 | 1 | 1 | 0 | 23,6 | 2 | 0 | 0 | 0 | 0,34 |
| 1401320 | 23,04 | 0 | 0 | 0 | 2 | 1 | 0 | 0 | 1 | 0 | 0 | 21,4 | 2 | 0 | 1 | 13 | 0,02 |
| 1401321 | 21,17 | 0 | 0 | 0 | 2 | 1 | 0 | 0 | 0 | 0 | 0 | 17,6 | 1 | 1 | 1 | 15 | 3,57 |
| 1401322 | 21,61 | 0 | 0 | 1 | 1 | 1 | 0 | 0 | 1 | 0 | 0 | 26,6 | 3 | 1 | 0 | 0 | 0,01 |
| 1401323 | 21,41 | 0 | 0 | 0 | 2 | 1 | 0 | 0 | 1 | 0 | 0 | 24,3 | 2 | 0 | 0 | 0 | -0,06 |
| 1401324 | 21,11 | 0 | 0 | 0 | 2 | 1 | 0 | 0 | 0 | 0 | 0 | 21,9 | 2 | 1 | 0 | 5 | 0,67 |
| 1401325 | 22,80 | 0 | 0 | 1 | 1 | 1 | 0 | 0 | 0 | 0 | 0 | 18,9 | 2 | 0 | 1 | 22 | -0,01 |
| 1401326 | 21,77 | 0 | 0 | 1 | 2 | 1 | 0 | 0 | 0 | 0 | 0 | 21,3 | 2 | 1 | 1 | 16 | 1,07 |
| 1401327 | 21,86 | 0 | 0 | 1 | 2 | 1 | 0 | 0 | 1 | 1 | 0 | 21,3 | 2 | 0 | 0 | 6 | -0,22 |
| 1401328 | 20,42 | 0 | 0 | 0 | 2 | 1 | 0 | 0 | 1 | 0 | 0 | 25,7 | 3 | 0 | 1 | 14 | -0,02 |
| 1401329 | 21,77 | 0 | 0 | 0 | 2 | 1 | 0 | 0 | 1 | 1 | 0 | 19,5 | 2 | 1 | 0 | 0 | 0,7 |
| 1401330 | 21,55 | 1 | 0 | 0 | 2 | 0 | 0 | 0 | 1 | 1 | 0 | 21,1 | 2 | 1 | 1 | 13 | 0,76 |
| 1401331 | 21,25 | 0 | 0 | 0 | 1 | 1 | 0 | 0 | 1 | 0 | 0 | 28,7 | 3 | 0 | 0 | 9 | -0,02 |
| 1401332 | 23,05 | 0 | 0 | 0 | 1 | 1 | 0 | 0 | 0 | 0 | 0 | 21,8 | 2 | 1 | 1 | 48 | 5,01 |
| 1401333 | 20,32 | 1 | 0 | 0 | 2 | 1 | 1 | 0 | 1 | 1 | 0 | 31,4 | 4 | 0 | 0 | 2 | 0,03 |
| 1401334 | 21,47 | 0 | 0 | 0 | 1 | 1 | 0 | 0 | 1 | 0 | 1 | 21,2 | 2 | 0 | 0 | 0 | 0 |
| 1401335 | 20,72 | 0 | 0 | 0 | 1 | 1 | 0 | 0 | 1 | 1 | 0 | 21,9 | 2 | 0 | 0 | 8 | 0,32 |
| 1401336 | 21,44 | 0 | 0 | 1 | 1 | 1 | 0 | 0 | 1 | 0 | 0 | 22,6 | 2 | 0 | 0 | 7 | -1,06 |
| 1401337 | 21,02 | 0 | 0 | 0 | 2 | 0 | 0 | 0 | 1 | 1 | 0 | 27,1 | 3 | 0 | 0 | 0 | -0,04 |
| 1401338 | 24,01 | 1 | 0 | 0 | 2 | 1 | 0 | 0 | 0 | 0 | 0 | 22,3 | 2 | 1 | 1 | 17 | 0,43 |
| 1401339 | 21,73 | 0 | 0 | 0 | 1 | 1 | 0 | 0 | 1 | 1 | 0 | 16,5 | 1 | 0 | 0 | 7 | 0 |
| 1401340 | 21,89 | 1 | 0 | 0 | 2 | 0 | 1 | 0 | 0 | 0 | 1 | 23,0 | 2 | 0 | 1 | 17 | -0,05 |
| 1401341 | 21,78 | 0 | 0 | 0 | 1 | 1 | 0 | 0 | 1 | 0 | 1 | 19,1 | 2 | 0 | 1 | 16 | 0,01 |
| 1401342 | 21,07 | 0 | 0 | 0 | 1 | 1 | 0 | 0 | 0 | 0 | 1 | 33,7 | 4 | 0 | 0 | 5 | -0,12 |
| 1401343 | 20,57 | 0 | 0 | 0 | 2 | 1 | 0 | 0 | 0 | 0 | 1 | 19,6 | 2 | 0 | 1 | 11 | -0,06 |
| 1401344 | 24,71 | 0 | 0 | 0 | 2 | 1 | 0 | 0 | 1 | 1 | 0 | 24,3 | 2 | 1 | 0 | 3 | 0,01 |
| 1401345 | 23,42 | 0 | 0 | 0 | 2 | 1 | 0 | 0 | 1 | 1 | 0 | 18,0 | 1 | 1 | 1 | 20 | 2,88 |
| 1401346 | 21,87 | 1 | 0 | 0 | 2 | 1 | 0 | 0 | 1 | 0 | 0 | 26,2 | 3 | 1 | 1 | 14 | 0,28 |
| 1401347 | 21,78 | 1 | 0 | 0 | 2 | 1 | 0 | 0 | 1 | 1 | 0 | 17,7 | 1 | 0 | 0 | 8 | 0,01 |
| 1401348 | 21,00 | 1 | 0 | 0 | 1 | 1 | 0 | 0 | 1 | 1 | 1 | 21,5 | 2 | 0 | 0 | 0 | -0,05 |
| 1401349 | 20,96 | 1 | 0 | 0 | 2 | 1 | 0 | 0 | 1 | 0 | 0 | 25,0 | 2 | 0 | 0 | 1 | -0,15 |
| 1401350 | 22,29 | 0 | 0 | 0 | 2 | 1 | 0 | 0 | 1 | 0 | 0 | 27,0 | 3 | 0 | 0 | 3 | 0,07 |
| 1401351 | 20,16 | 0 | 0 | 0 | 2 | 0 | 0 | 0 | 1 | 1 | 1 | 20,3 | 2 | 0 | 0 | 3 | -0,01 |
| 1401352 | 21,39 | 0 | 0 | 0 | 2 | 1 | 0 | 0 | 1 | 0 | 1 | 19,8 | 2 | 0 | 0 | 7 | -0,01 |
| 1401353 | 22,15 | 1 | 0 | 0 | 2 | 0 | 0 | 0 | 1 | 1 | 1 | 19,4 | 2 | 0 | 0 | 4 | -0,4 |
| 1401354 | 21,50 | 1 | 0 | 0 | 1 | 1 | 0 | 0 | 1 | 1 | 1 | 16,6 | 1 | 0 | 0 | 7 | -0,08 |
| 1401355 | 21,45 | 0 | 0 | 0 | 2 | 1 | 0 | 0 | 1 | 1 | 1 | 20,1 | 2 | 0 | 0 | 9 | 0,02 |
| 1401356 | 22,19 | 0 | 0 | 1 | 1 | 1 | 0 | 0 | 1 | 1 | 0 | 20,5 | 2 | 0 | 0 | 0 | 0 |
| 1401357 | 21,45 | 0 | 0 | 0 | 1 | 1 | 0 | 0 | 1 | 1 | 1 | 31,3 | 4 | 0 | 1 | 12 | -0,05 |
| 1401358 | 20,97 | 0 | 0 | 1 | 1 | 1 | 0 | 0 | 0 | 1 | 1 | 17,1 | 1 | 0 | 0 | 5 | 0,02 |
| 1401359 | 22,46 | 1 | 0 | 1 | 1 | 1 | 0 | 0 | 1 | 1 | 0 | 21,9 | 2 | 0 | 0 | 9 | -0,17 |

| | | | | | | | | | | | | | | | | | |
|---|---|---|---|---|---|---|---|---|---|---|---|---|---|---|---|---|---|
| 1401360 | 21,67 | 0 | 0 | 0 | 2 | 1 | 0 | 0 | 0 | 0 | 0 | 24,5 | 2 | 0 | 0 | 9 | 0,01 |
| 1401361 | 22,05 | 0 | 0 | 0 | 1 | 1 | 0 | 0 | 1 | 1 | 0 | 20,7 | 2 | 0 | 1 | 14 | 0 |
| 1401362 | 21,76 | 0 | 0 | 1 | 2 | 1 | 0 | 0 | 1 | 1 | 1 | 31,4 | 4 | 0 | 0 | 2 | 0,3 |
| 1401363 | 20,56 | 1 | 0 | 0 | 2 | 1 | 1 | 0 | 1 | 1 | 0 | 23,2 | 2 | 0 | 1 | 14 | 0 |
| 1401364 | 21,70 | 0 | 0 | 1 | 1 | 0 | 0 | 0 | 1 | 1 | 0 | 19,8 | 2 | 0 | 0 | 6 | 0,01 |
| 1401365 | 21,56 | 0 | 0 | 1 | 2 | 1 | 0 | 0 | 1 | 1 | 0 | 18,7 | 2 | 0 | 0 | 9 | 0,23 |
| 1401366 | 21,02 | 0 | 0 | 0 | 2 | 1 | 0 | 0 | 1 | 1 | 0 | 16,6 | 1 | 0 | 0 | 2 | 0,02 |
| 1401367 | 21,44 | 0 | 0 | 0 | 2 | 1 | 0 | 0 | 1 | 1 | 0 | 21,7 | 2 | 0 | 0 | 3 | 0,1 |
| 1401368 | 21,13 | 0 | 0 | 0 | 2 | 1 | 0 | 0 | 1 | 1 | 0 | 24,9 | 2 | 0 | 0 | 3 | 0,01 |
| 1401369 | 22,03 | 1 | 0 | 1 | 2 | 1 | 0 | 0 | 1 | 1 | 0 | 29,0 | 3 | 1 | 1 | 15 | 3,73 |
| 1401370 | 21,58 | 0 | 0 | 0 | 2 | 1 | 0 | 0 | 1 | 1 | 0 | 23,9 | 2 | 0 | 0 | 0 | 0,02 |
| 1401371 | 21,53 | 1 | 0 | 0 | 2 | 1 | 0 | 0 | 1 | 1 | 0 | 23,7 | 2 | 0 | 0 | 0 | -0,01 |
| 1401372 | 23,36 | 0 | 1 | 0 | 1 | 1 | 0 | 0 | 1 | 1 | 1 | 23,0 | 2 | 0 | 0 | 0 | 0,02 |
| 1401374 | 21,54 | 0 | 0 | 0 | 2 | 1 | 0 | 0 | 1 | 1 | 1 | 21,7 | 2 | 0 | 1 | 20 | 0 |
| 1401375 | 23,00 | 0 | 0 | 0 | 2 | 1 | 0 | 0 | 1 | 1 | 0 | 22,1 | 2 | 0 | 0 | 4 | -0,05 |
| 1401376 | 20,52 | 0 | 0 | 1 | 2 | 1 | 0 | 0 | 1 | 1 | 0 | 17,3 | 1 | 0 | 1 | 11 | -0,01 |
| 1401377 | 20,76 | 0 | 0 | 1 | 2 | 1 | 0 | 0 | 1 | 1 | 1 | 23,3 | 2 | 0 | 1 | 23 | -1,02 |
| 1401378 | 21,50 | 0 | 0 | 0 | 2 | 1 | 0 | 0 | 1 | 0 | 1 | 21,7 | 2 | 0 | 0 | 8 | -0,32 |
| 1401379 | 21,58 | 1 | 0 | 1 | 2 | 1 | 1 | 0 | 1 | 1 | 1 | 29,0 | 3 | 0 | 0 | 6 | 0 |
| 1401380 | 22,90 | 1 | 0 | 0 | 2 | 1 | 0 | 0 | 1 | 1 | 0 | 20,9 | 2 | 0 | 0 | 0 | 0 |
| 1401381 | 21,65 | 1 | 0 | 0 | 2 | 1 | 1 | 0 | 1 | 0 | 0 | 18,7 | 2 | 0 | 0 | 3 | 0 |
| 1401382 | 22,60 | 1 | 1 | 0 | 1 | 1 | 0 | 0 | 1 | 1 | 1 | 22,1 | 2 | 0 | 0 | 0 | 0,03 |
| 1401385 | 22,32 | 0 | 1 | 0 | 2 | 1 | 0 | 0 | 1 | 1 | 1 | 17,9 | 1 | 0 | 0 | 4 | 0,05 |
| 1401386 | 22,26 | 0 | 1 | 0 | 1 | 1 | 0 | 0 | 1 | 1 | 0 | 24,7 | 2 | 0 | 0 | 9 | 0,29 |
| 1401387 | 21,67 | 0 | 1 | 1 | 1 | 1 | 0 | 0 | 1 | 0 | 0 | 18,5 | 1 | 0 | 0 | 6 | -0,01 |
| 1401388 | 22,89 | 0 | 1 | 1 | 1 | 1 | 0 | 0 | 1 | 1 | 1 | 26,8 | 3 | 0 | 0 | 5 | 0,04 |
| 1401389 | 21,89 | 0 | 1 | 0 | 1 | 1 | 0 | 0 | 0 | 0 | 1 | 18,0 | 1 | 0 | 1 | 14 | 0,04 |
| 1401390 | 23,06 | 1 | 1 | 1 | 1 | 1 | 0 | 0 | 1 | 1 | 1 | 22,8 | 2 | 0 | 0 | 1 | 0,05 |
| 1401392 | 22,81 | 1 | 1 | 0 | 1 | 1 | 0 | 0 | 1 | 1 | 1 | 32,3 | 4 | 0 | 0 | 4 | 0,07 |
| 1401393 | 22,39 | 0 | 1 | 1 | 1 | 1 | 0 | 0 | 1 | 1 | 0 | 20,7 | 2 | 0 | 0 | 0 | 0 |
| 1401395 | 23,21 | 1 | 1 | 1 | 1 | 0 | 0 | 0 | 0 | 0 | 0 | 24,8 | 2 | 0 | 0 | 9 | 0,05 |
| 1401396 | 22,60 | 0 | 1 | 0 | 2 | 1 | 0 | 0 | 1 | 1 | 1 | 27,4 | 3 | 0 | 1 | 10 | -0,39 |
| 1401398 | 23,93 | 1 | 1 | 0 | 1 | 1 | 1 | 0 | 1 | 1 | 0 | 24,4 | 2 | 0 | 1 | 11 | 0 |
| 1401399 | 24,18 | 0 | 1 | 1 | 2 | 0 | 0 | 0 | 0 | 0 | 0 | 24,7 | 2 | 1 | 1 | 20 | 0,72 |
| 1401400 | 22,02 | 0 | 1 | 0 | 2 | 1 | 0 | 0 | 1 | 1 | 0 | 22,4 | 2 | 0 | 1 | 19 | 0,16 |
| 1401401 | 23,37 | 1 | 1 | 0 | 2 | 1 | 1 | 0 | 1 | 1 | 1 | 23,6 | 2 | 0 | 1 | 13 | -0,02 |
| 1401402 | 22,35 | 0 | 1 | 0 | 2 | 1 | 0 | 0 | 1 | 1 | 0 | 18,6 | 2 | 0 | 0 | 0 | 0 |
| 1401403 | 22,73 | 0 | 1 | 0 | 2 | 0 | 0 | 0 | 1 | 1 | 1 | 23,7 | 2 | 0 | 0 | 0 | 0,02 |
| 1401404 | 23,12 | 0 | 1 | 1 | 2 | 1 | 0 | 0 | 1 | 0 | 0 | 30,2 | 4 | 0 | 1 | 16 | 0,06 |
| 1401405 | 22,09 | 0 | 1 | 0 | 2 | 1 | 0 | 0 | 1 | 1 | 1 | 24,3 | 2 | 0 | 0 | 0 | 0,01 |
| 1401406 | 22,10 | 0 | 1 | 1 | 1 | 1 | 0 | 0 | 1 | 1 | 0 | 26,7 | 3 | 0 | 0 | 2 | -0,01 |
| 1401407 | 22,70 | 0 | 1 | 0 | 2 | 1 | 0 | 0 | 1 | 1 | 1 | 19,5 | 2 | 0 | 0 | 0 | 0 |
| 1401408 | 22,86 | 0 | 1 | 1 | 1 | 1 | 0 | 0 | 1 | 1 | 1 | 21,9 | 2 | 0 | 0 | 6 | 0 |
| 1401409 | 22,52 | 0 | 1 | 0 | 1 | 1 | 0 | 0 | 1 | 0 | 1 | 20,1 | 2 | 0 | 0 | 9 | 0,03 |

| | | | | | | | | | | | | | | | | | |
|---|---|---|---|---|---|---|---|---|---|---|---|---|---|---|---|---|---|
| 1401410 | 23,25 | 0 | 1 | 0 | 1 | 0 | 0 | 0 | 1 | 1 | 0 | 26,8 | 3 | 1 | 1 | 17 | 1,46 |
| 1401411 | 21,91 | 0 | 1 | 0 | 1 | 1 | 0 | 0 | 1 | 0 | 0 | 23,9 | 2 | 0 | 0 | 2 | 0 |
| 1401412 | 23,20 | 0 | 1 | 0 | 2 | 1 | 0 | 0 | 1 | 1 | 0 | 23,9 | 2 | 0 | 1 | 13 | 0,01 |
| 1401413 | 22,85 | 0 | 1 | 0 | 1 | 1 | 0 | 0 | 1 | 1 | 1 | 22,9 | 2 | 0 | 0 | 9 | -0,01 |
| 1401414 | 22,74 | 0 | 1 | 1 | 1 | 1 | 0 | 0 | 1 | 1 | 0 | 20,1 | 2 | 1 | 1 | 45 | 1,63 |
