# Supplementary material for: Tuberculin skin test and Interferon-gamma release assay agreement, and associated factors with latent tuberculosis infection, in medical and nursing students in Bandung, Indonesia
Source: PLoS One. 2024 Mar 18;19(3):e0299874. doi: 10.1371/journal.pone.0299874 (PMC10947906; doi:10.1371/journal.pone.0299874)
Supplement: S1 Questionnaire — (PDF) [file pone.0299874.s002.pdf]

DATA COLLECTION FORM AT BASELINE

1	Date of interview	|__|__|/|__|__|/|__|__| Day/Month/Year
2	Interviewer initials	|__|__|
3	Start time of interview	|__|__|:|__|__|
**DEMOGRAPHIC DETAILS**		
4	Participant number	**PARTICIPANT STICKER**
5	Date of birth	|__|__|/|__|__|/|__|__| Day/Month/Year
6	Age	|__|__|Years
7	Gender	|__| 1 = Male, 0 = Female
8	Student type	|__| 1 = Medical, 2 = Nursing
9	Month and year of entry into internship program	|__|__|/|__|__| Month/Year
10	Where do you stay	|__| 1 = Family housing, 2 = Student housing
11	Ethnicity	|__| 1 = Sundanese, 2 = Javanese, 3 = Batak, 4 = Betawi, 5 = Madurese, 6 = Minangkabau, 7 = Acehnese, 8 = Balinese, 9 = Chinese, 10 = Other (please state)<br>________________________
**MEDICAL HISTORY**		
12	BCG vaccination	|__| 1 = Vaccinated, 0 = Not vaccinated,
13	Do you have a BCG scar? (can I please see it?)	|__| 1 = Yes, 0 = No
14	Have you ever been diagnosed with HIV?	|__| 1 = Yes, 0 = No
15	Do you have any other immunocompromised condition?<br>*(read the options to the participant)*	|__| 1 = Diabetes, 2 = Systemic Lupus Erythematosus, 3 = Renal failure, 4 = Transplant anti-rejection therapy, 5 = TNFα inhibitor therapy, 6 = Other immune-suppressive condition or therapy (steroid), 7 = None
16	Do you smoke cigarettes?<br>*(read the options to the participant)*	|__| 1 = Yes current , 2 = Ex-Smoker, 3 = Never
17	If current or ex-smoker	Age started: |__|years

<table>
<tr><td></td><td></td><td>Packs/day: |__|</td></tr>
<tr><td>18</td><td>If ex-smoker</td><td>Age stopped: |__|years</td></tr>
<tr><td>19</td><td>Do you consume alcohol?</td><td>|__|
1 = Yes, 0 = No</td></tr>
<tr><td colspan="3">TB EXPOSURE</td></tr>
<tr><td>20</td><td>Have you ever been involved in other training/project in hospital/clinic prior to study enrolment?</td><td>|__|
1 = Yes, 0 = No</td></tr>
<tr><td>21</td><td>If yes, any direct contact with TB patient or participation in sputum collection or examination?</td><td>|__|
1 = Yes, 0 = No</td></tr>
<tr><td>22</td><td>If yes, state the location</td><td>|__|
1 = DOTS clinic, 2 = Isolation ward, 3 = Laboratory, 4 = Pulmonary ward, 5 = Other (please state) ___________</td></tr>
<tr><td>23</td><td>When?</td><td>|__|__|Months ago
|__|__|Years ago</td></tr>
<tr><td>24</td><td>Have you ever had direct contact with family or friends who have been diagnosed with TB?</td><td>|__|
1 = Yes, 0 = No</td></tr>
<tr><td>25</td><td>If yes, how many years/months ago?</td><td>|__|__|Months ago
|__|__|Years ago</td></tr>
<tr><td colspan="3">ACTIVE TB SCREENING</td></tr>
<tr><td>26</td><td>Dry cough</td><td>|__| 1 = Yes, 0 = No
Duration: |__|__| days or |__|__| weeks</td></tr>
<tr><td>27</td><td>Productive cough</td><td>|__| 1 = Yes, 0 = No
Duration: |__|__| days or |__|__| weeks</td></tr>
<tr><td>28</td><td>Cough at night</td><td>|__| 1 = Yes, 0 = No
Duration: |__|__| days or |__|__| weeks</td></tr>
<tr><td>29</td><td>Pain in the chest</td><td>|__| 1 = Yes, 0 = No
Duration: |__|__| days or |__|__| weeks</td></tr>
<tr><td>30</td><td>Coughing up blood or sputum</td><td>|__| 1 = Yes, 0 = No
Duration: |__|__| days or |__|__| weeks</td></tr>
<tr><td>31</td><td>Night sweats</td><td>|__| 1 = Yes, 0 = No
Duration: |__|__| days or |__|__| weeks</td></tr>
<tr><td>32</td><td>Weakness or fatigue</td><td>|__| 1 = Yes, 0 = No
Duration: |__|__| days or |__|__| weeks</td></tr>
<tr><td>33</td><td>Weight loss</td><td>|__| 1 = Yes, 0 = No
Duration: |__|__| days or |__|__| weeks</td></tr>
<tr><td>34</td><td>No appetite</td><td>|__| 1 = Yes, 0 = No
Duration: |__|__| days or |__|__| weeks</td></tr>
<tr><td>35</td><td>Fever</td><td>|__| 1 = Yes, 0 = No
Duration: |__|__| days or |__|__| weeks</td></tr>
<tr><td>36</td><td>Visible Mass in Neck</td><td>|__| 1 = Yes, 0 = No</td></tr>
</table>

		Duration: |__|__| days or |__|__| weeks
37	End time of interview	|__|__|:|__|__|
**BODY MASS INDEX**		
38	Height	|__|__|__| cm
39	Weight	|__|__|__|. |__|__| kg
**IGRA TEST**		
40	Date of blood drawing for IGRA	|__|__|/|__|__|/|__|__| Day/Month/Year
41	IGRA test result qualitative	|__| 1 = Positive, 0 = Negative, 2 = Intermediate
**TUBERCULIN SKIN TEST**		
42	Date of TST administered	|__|__|/|__|__|/|__|__| Day/Month/Year
43	Date of TST read	|__|__|/|__|__|/|__|__| Day/Month/Year
44	TST Result (diameter)	|__|__| mm
45	TST Result	|__| 1 = Positive, 0 = Negative
