# Supplementary material for: Tuberculin skin test and Interferon-gamma release assay agreement, and associated factors with latent tuberculosis infection, in medical and nursing students in Bandung, Indonesia
Source: PLoS One. 2024 Mar 18;19(3):e0299874. doi: 10.1371/journal.pone.0299874 (PMC10947906; doi:10.1371/journal.pone.0299874)
Supplement: Attachment — Submitted filename: Response to Reviewers PLOS ONE.docx [file pone.0299874.s003.docx]

**1 February 2024**

**Mao-Shui Wang**

**Academic Editor**

**PLOS ONE**

**Dear Dr Wang,**

We are pleased to resubmit our manuscript entitled “Tuberculin skin test and Interferon-gamma release assay agreement, and associated factors with latent tuberculosis infection, in medical and nursing students in Bandung, Indonesia” for consideration for publication in PLOS ONE.

We would like thank the editor and reviewers for their high quality and constructive review and for careful reading of our manuscript. In this revised version of the manuscript, we have addressed all comments raised by the editor and reviewers. Changes to the manuscript are indicated by track changes. A detailed point-by point response to each of the editor and reviewers’ point follows.

**Journal requirements (Editor):**

When submitting your revision, we need you to address these additional requirements.

1. Please ensure that your manuscript meets PLOS ONE's style requirements, including those for file naming. The PLOS ONE style templates can be found at

<https://journals.plos.org/plosone/s/file?id=wjVg/PLOSOne_formatting_sample_main_body.pdf> and

<https://journals.plos.org/plosone/s/file?id=ba62/PLOSOne_formatting_sample_title_authors_affiliations.pdf>

**Response:** The manuscript has been revised to meet PLOS ONE’s style requirements.	This includes Tables 1, 2, 3, and 4.

2. We noticed you have some minor occurrence of overlapping text with the following previous publication(s), which needs to be addressed:

- <https://doi.org/10.1093/trstmh/trab038>

In your revision ensure you cite all your sources (including your own works), and quote or rephrase any duplicated text outside the methods section. Further consideration is dependent on these concerns being addressed.

**Response:** The results and discussion section have been revised. Please see lines 162-176 in the results section, lines 240-242, and lines 264-266 in the discussion.

3. We note that the grant information you provided in the ‘Funding Information’ and ‘Financial Disclosure’ sections do not match.

When you resubmit, please ensure that you provide the correct grant numbers for the awards you received for your study in the ‘Funding Information’ section.

**Response:** The Funding Information has been corrected on the website and in the revised manuscript. We added the information about the cost for publication funded by Universitas Padjadjaran.

4. In the online submission form, you indicated that [The datasets used and/or analysed during the current study are available from the corresponding author on reasonable request].

All PLOS journals now require all data underlying the findings described in their manuscript to be freely available to other researchers, either 1. In a public repository, 2. Within the manuscript itself, or 3. Uploaded as supplementary information.

This policy applies to all data except where public deposition would breach compliance with the protocol approved by your research ethics board. If your data cannot be made publicly available for ethical or legal reasons (e.g., public availability would compromise patient privacy), please explain your reasons on resubmission and your exemption request will be escalated for approval.

**Response:** This is now been corrected. All relevant data are within the manuscript and its Supporting Information files (S1).

5. Please review your reference list to ensure that it is complete and correct. If you have cited papers that have been retracted, please include the rationale for doing so in the manuscript text, or remove these references and replace them with relevant current references. Any changes to the reference list should be mentioned in the rebuttal letter that accompanies your revised manuscript. If you need to cite a retracted article, indicate the article’s retracted status in the References list and also include a citation and full reference for the retraction notice.

**Response:** We have reviewed all references and none of them have been retracted. However, we delete one reference “Menzies RI. Tuberculin Skin Testing (2000) In Reichman, L.B. and Hershfield, E.S., Eds., Tuberculosis: A Comprehensive International Approach, Second Edition, Revised and Expanded, Marcel Dekker, New York, 279-322.” Due to unavailable article.

**Reviewer #1: The manuscript presented interesting findings.**

Minor comments:

1. The authors noted that the current study was a part of a preceding study (10.1093/trstmh/trab038). In that earlier study, a total of 379 individuals underwent analysis, with 70 testing positive for IGRA. In the present study, the authors initially identified 301 individuals and subsequently analyzed 266, of whom 43 tested positive for IGRA. Please provide detailed clarification on the inclusion and exclusion processes. A flow diagram illustrating subject recruitment would be beneficial in elucidating the included patients, if deemed necessary

**Response:** Thank you for the comment. In the preceding study, we included all students entering their clinical program at two different times, which were in August 2016 and January 2017, and tested them with IGRA. In this present study, we only included students in the January 2017 batch who were also tested by TST, not only IGRA. Therefore the number of students who underwent analysis in this study was only 266 subjects. We have added information about this (Lines 107-108). We also have added a figure of subject recruitment as Fig 1. The scatter graphs showing correlations between quantitative IGRA and TST test results have now become Fig 2.

2. Is the questionnaire available? I would recommend attach it as a supplementary file

**Response:** Yes, the questionnaire is now attached as a supplementary file (S2).

3. Line 122-123: Who interpret the TST? Was it the same nurse?

**Response:** Yes, it was the same nurse. We have added the information in line 125.

4. Table 1: The majority of the study population came from “ethnicities other than Sundanese”. Can this be specified? as this was the majority (perhaps the top three).

**Response**: We have added the top three ethnicities in Table 1.

5. Table 4: Please include columns for "negative IGRA" and "negative TST." In the columns "positive IGRA" and "positive TST," what were the denominators used for percentage calculation? For instance, the proportion of males with a positive IGRA was 9 individuals, and the percentage in parentheses was 12.7. How did the authors calculate 12.7% as 9/265 (all included individuals) equal to 3.4%, and 9/43 (positive IGRA only) equal to 20.9%? Were these calculated on a "row" basis? If so, the table should be revised for better clarity.

**Response:** Yes, the calculation was on a “row” basis. As an example: the proportion of males with a positive IGRA was 12.7% which was 9/71. We have added columns for “negative IGRA” and “negative TST” in Table 4 to clarify. We also added the information on the percentages by row in the footnote. Since one student had IGRA indeterminate result, we only analysed 265 students for IGRA, and 266 students for TST.

6. One student with an indeterminate IGRA result (Line 171-172) was not included in the agreement and risk factor analysis. Please specify the TST result.

**Response:** We have added the TST result (positive) for this student (line 200 now).

7. Line 225-26: “The small number of our study subjects could also make the estimate unstable”. What do the authors mean by "unstable"? How many additional samples do the authors anticipate, and what is the rationale for this? I would prefer to delete this sentence instead.

**Response:** Thank you, we have deleted this sentence.

8. (Line 232) the authors are expected to elaborate more on possible NTM in the population as this can affect the false-positive TST (i.e., possible [high?] exposure in the medical/nursing academic environment as well).

**Response:** We have added information on possible high NTM in the population (Line 294).

**Reviewer #2: The study explains the association between TST and IGRA performed on Medical students and nurses before commencement of clinical rotation. I have following minor comments.**

1. The study was done almost 6 yrs ago that is Jan-Feb 2017. I do not know the Journal policy it accepts more than 5 yrs old study or not.

**Response:** We think that this study is still relevant, even though more than 5 years old study. We wanted to get the main paper published first (January 2022) before submitting this one. For both manuscripts there were delays due to Covid-19 in manuscript completion and also in finding reviewers once submitted. This current manuscript, for example, was previously submitted to another journal and unfortunately, after extensive efforts by the review selection editors, they were not able to obtain the required number of reviewers and therefore withdrew the submission. We hope that this situation can be understood.

2. In introduction, please mention the incidence and prevalence of TB in Indonesia with reference.

**Response:** We have added the number of total TB incidence and incidence rate in Indonesia (lines 90-91).

3. Line 122. Please write clearly the time for reading, 48 or 72 hrs as it can produce a false negative result.

**Response:** We have clarified the time for reading (line 125).

We thank the editor and reviewers for their appreciation of our work.

We hope the revised manuscript will be appropriate and will be reconsidered for publication in PLOS ONE.

Yours sincerely,

Lika Apriani, PhD

On behalf of all authors
